# Dog Bite Histories and Response to Incidents in Canine Rabies-Enzootic KwaZulu-Natal, South Africa

**DOI:** 10.1371/journal.pntd.0002059

**Published:** 2013-04-04

**Authors:** Melinda Hergert, Louis H. Nel

**Affiliations:** 1 Paraclinical Sciences Department, Faculty of Veterinary Science, University of Pretoria, Onderstepoort, South Africa; 2 Department of Microbiology and Plant Pathology, Faculty of Natural and Agricultural Sciences, University of Pretoria, Pretoria, South Africa; The Global Alliance for Rabies Control, United States of America

## Abstract

The objective of this paper is to report evaluated observations from survey records captured through a cross-sectional observational study regarding canine populations and dog owners in rabies enzootic KwaZulu-Natal province, South Africa. Our aim was to evaluate respondent knowledge of canine rabies and response to dog bite incidents towards improved rabies control. Six communities consisting of three land use types were randomly sampled from September 2009 to January 2011, using a cluster design. A total of 1992 household records were analyzed using descriptive statistics and regression modeling to evaluate source of rabies knowledge, experiences with dog bites, and factors affecting treatment received within respective households that occurred within the 365 day period prior to the surveys. 86% of the population surveyed had heard of rabies. Non-dog owners were 1.6 times more likely to have heard of rabies than dog owners; however, fear of rabies was not a reason for not owning a dog. Government veterinary services were reported most frequently as respondent source of rabies knowledge. Nearly 13% of households had a member bitten by a dog within the year prior to the surveys with 82% of the victims visiting a clinic as a response to the bite. 35% of these clinic visitors received at least one rabies vaccination. Regression modeling determined that the only response variable that significantly reflected the likelihood of a patient receiving rabies vaccination or not was the term for the area surveyed. Overall the survey showed that most respondents have heard of dog associated rabies and seek medical assistance at a clinic in response to a dog bite regardless of offending dog identification. An in-depth study involving factors associated within area clinics may highlight the area dependency for patients receiving rabies post exposure prophylaxis shown by this model.

## Author Summary

Canine rabies has been enzootic to KwaZulu-Natal province, South Africa since the mid-1970's. Vaccination requirements for domestic species and animal control laws enforced in industrialized countries frequently eliminate the need for rabies post exposure prophylaxis (PEP) when an animal bite occurs. Rabies deaths in Africa are frequently linked to poverty and ignorance resulting in a lack of urgency for PEP in an environment where less than 70% of the domestic dog population is vaccinated against the disease. The results presented here are part of a larger canine ecology study conducted in KwaZulu-Natal from September 2009 through January 2011. The six surveyed areas consisted of three land use types: three rural villages, two urban townships and one peri-urban township. The findings show that although a large portion of the population has awareness of rabies, there is a lack of understanding in the response to dog bites. Regression modeling of data suggests that there is an effect of area upon the result of a bite victim receiving PEP as part of treatment. Detailed retrospective study of dog bite incidence and an introspective study of clinics and treatment centers within the province may help explain the results found in this study.

## Introduction

Rabies kills tens of thousands of people in developing countries each year, and it is estimated that almost half of global rabies incidences occur in Africa [1]–[2]. However, one major factor compounding the problems of rabies is a high probability of disease underreporting. Studies in Tanzania, for example, indicated that there are ten cases for every one officially reported [1]. Once clinical signs of encephalitis become apparent, human rabies is virtually untreatable [3]. Although there has been at least one bona fide case of survival using intensive care treatment, much remains to be understood about factors determining the outcome of such treatment procedures while the required facilities and cost of procedure put such interventions outside the reach of those countries where dog and human rabies is most prevalent [4]. Reliance on proper wound management, and timely post exposure prophylaxis (PEP) [appropriate administration of vaccine and immunoglobulin], is crucial to the prevention of human rabies in exposed persons.

From the above perspective, rabies deaths in Africa are linked to ignorance and poverty. People from rural areas and young children, lacking knowledge of rabies and thus the requirement and urgency of PEP, are most frequently affected [5]. In just one example, from a relatively progressive African state, viz. South Africa, it was shown that half of the laboratory confirmed cases from 2008 did not seek any medical intervention after dog bite exposure [6]. Although rabies PEP is free of charge for bite victims in South Africa, the full post exposure treatment with vaccine and immunoglobulin G costs the South African health system more than USD $152 per individual [7].

**Figure 1:**
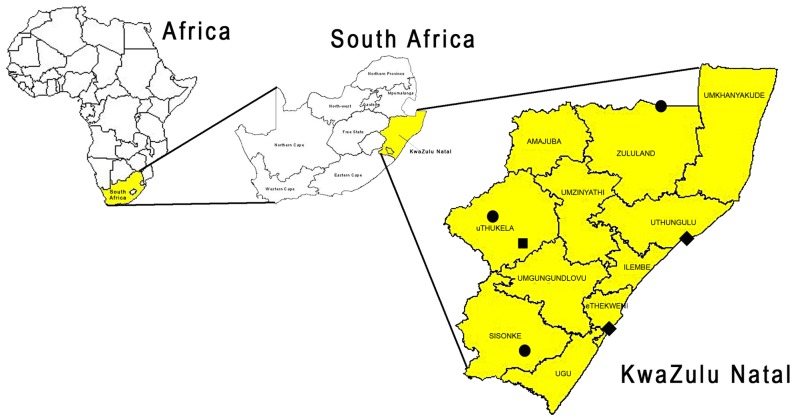
Geographical location of KwaZulu-Natal with the six study areas indicated. Black square = Wembezi (peri-urban, rabies free), black diamond = Umlazi and Esikhawini (urban, rabies enzootic), black circle = Ixopo, Pongola and St. Chad's (rural, rabies enzootic).

KwaZulu-Natal (KZN), one of nine provinces, is located on the east coast of South Africa (Figure 1) with an area of 94,361 km^2^ and a human population last estimated at 10,819,130 with a growth rate of 1.2% for all races of people [8]. KZN contains just over 21% of the total human population for South Africa despite the province being only 7.7% of the country's land mass. Over 84% of the population is black African, mostly of Zulu cultural origin [8]. It is thought that canine rabies spread to KZN from adjacent Mozambique during the 1960's. Although the disease was then eradicated through the use of mass vaccination campaigns and dog control, rabies was reintroduced in the mid 1970's and has been enzootic to KZN ever since [9]. Historically, most of the human rabies cases in South Africa over past decades have been from KZN. Many dogs in South Africa are not immunized against rabies despite laws mandating vaccination, and KZN is no exception. High dog population turnover, lax enforcement of government regulations and interruptions in vaccination campaigns are all likely factors that contribute to low rabies immunization coverage. In this regard, rabies does not generally appear to enjoy appropriate public health priority in African countries. Poor reporting and poor surveillance, resulting in an apparent lack of political commitment to rabies control, seems to be common practice. In this study it was our objective to better quantify issues such as the above, for the particular region of KZN. Here we analyze the responses to dog bites in an area that has been dog rabies-enzootic for decades, and where control has been attempted for an equal period of time. A better understanding of the societies and practices involved, including knowledge and awareness, would be crucial in improving a disease situation that has been ongoing for the past 40 years. As part of a comprehensive rabies control program, we have queried a representative sample of several population segments of the KZN province about rabies knowledge, interest in enforcement of animal control laws and in community based surveillance.

## Materials and Methods

### Study area and sampling procedure

From September 2009 through January 2011, household surveys were conducted in six different communities across KZN province, covering three land use types: rural, urban and peri-urban (Figure 1). Distribution of the 1992 households completing the surveys was 52% rural, 33% urban and 15% peri-urban. Rabies was enzootic in all areas, with the exception of the peri-urban community of Wembezi. Affluent urban and suburban areas where people keep dogs in confined spaces most likely have lower rabies risk due to fewer affective contacts between animals and easier access to veterinary services and were therefore excluded [10]. Poorer urban townships and rural villages most frequently represent the areas from where canine rabies is reported (KZNDAERD unpublished data). The study areas were selected with the assistance of the KZN Department of Agriculture and Environmental Affairs and Rural Development (KZNDAERD), Veterinary Services division.

Simple random sampling and systematic surveys are difficult in developing countries due to logistical and sometimes adverse cultural reasons [11]. Random sampling using a cluster or ‘area’ design was used because homesteads in rural areas are not numbered and informal housing settlements within townships frequently are arranged haphazardly [12].

### Questionnaire interviews

Based upon World Health Organization guidelines [13] the questionnaires were composed of two parts; a household survey for collecting demographics and community opinions, and an individual dog survey for descriptive statistics of the owned dog population. Though the primary objective of the surveys was to gain provincial dog demographics, respondents were asked exploratory questions regarding their knowledge of rabies, histories of dog bites and response to those incidents in consideration of possible future studies. Respondents were also queried about their interest in animal-law enforcement, animal ownership regulations and community based surveillance. The surveys were translated into isiZulu and then back translated to English before being piloted in a township with similar human demographics and a history of canine rabies. The survey tool was refined prior to use in this study and the final questionnaires were well received by both the surveyors and the target population with no further improvements or modifications required. KZNDAERD Animal Health Technicians and students, Department of Health workers, Environmental Health workers and SPCA employees were trained to perform the surveys. Surveyors were instructed to introduce themselves to household respondents and explain the purpose of the questionnaire prior to asking their permission to carry out the survey. Surveyors wore name tags which had ‘Rabies Surveillance Team’ printed in large block letters with a bright red border and the Departmental insignia as an identification aid. Prior permission to conduct the surveys had been sought from municipal counselors. All interviews were conducted between the hours of 9 am and 3 pm.

### Data analysis

The data from each area was entered into a Microsoft Excel spreadsheet and then imported into SAS version 9.3 (SAS Institute, Inc., Cary, North Carolina, USA). Descriptive statistics were generated, and cross tabulations calculating Pearson's Chi Square (χ^2^) were performed in tests of association. A logistic regression model was built using SAS to predict outcomes of human dog bite victims receiving rabies PEP [14].

### Ethics statement

The study was approved by the University of Pretoria, Veterinary Faculty Research Committee at Onderstepoort campus. An application for the non-experimental use of animals was approved by the Animal Use and Care Committee from the University of Pretoria. Interviewed subjects were provided informed consent orally in their native language as was stated in the research proposal approved by the Research Committee. The purpose of the survey interview was explained to each participant by the interviewer, who could either accept or decline to participate in the survey. If the respondent declined to be interviewed it was marked at the top of the survey form, whereas those who agreed to be interviewed had a third party witness to this verbal agreement, and the interview was continued.

## Results

### Questionnaire interviews

A total of 1992 households consisting of 13,756 people (range 1–34, median = 6) completed the surveys within the three targeted community types. Surveys were answered by a person defined as head of the household in 68% (1361/1992) of the cases across the province (range 63–76%). The sex of the respondent was not recorded. Of the remaining cases, 11 comprised interviews with children under the age of fourteen years of age in the presence of an older relative who consented to the child answering questions. Another 183 children over the age of fourteen years were interviewed at homes where adults were not present. In 435 surveys, an adult other than the head of the household responded to interview questions. In 2 cases the category of the respondent was missing.

### Knowledge of rabies

**Figure 2:**
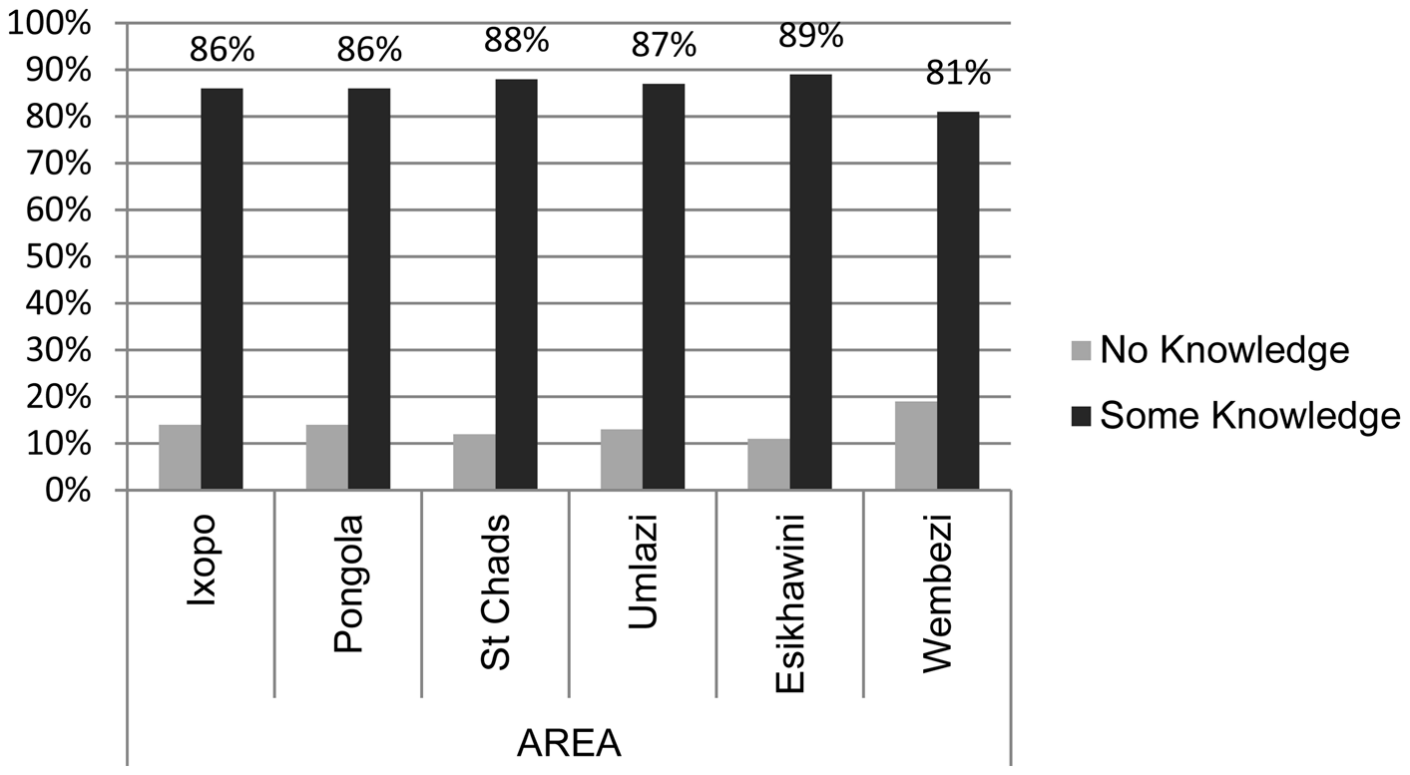
Respondent knowledge of rabies by area surveyed (n = 1990). Responses were recorded as either ‘no’ or ‘some’ knowledge of rabies. Areas are defined as: (Rabies enzootic rural = Ixopo, Pongola, St. Chad's; rabies enzootic urban = Umlazi, Esikhawini; rabies free peri-urban = Wembezi).

Eighty-six percent of the population (1716/1992) surveyed across the province had heard of the disease called rabies (Figure 2). No attempt was made to evaluate the individual's depth of rabies knowledge. Some respondents stated that they did not truly know the source of rabies or how to prevent it. However, it was clear that some respondents knew that vaccination of dogs was important to the safety of people in the community based upon their remarks.

There was no significant relationship found between area surveyed and respondent knowledge of rabies (χ^2 = ^10.864, df = 5, *p = *0.541). When surveyed areas were grouped by land use, 81% of the peri-urban society had some knowledge of rabies, whereas 87% of rural and 88% of urban citizens had knowledge of rabies. Surprisingly, non-dog owners were 1.6 times more likely to have heard of rabies compared with dog owners. No respondents stated that fear of rabies infection was a reason for not owning a dog.

### Source of knowledge

Persons who responded that they did have some knowledge of rabies were further queried as to the source of their knowledge. Government Veterinary Services Animal Health Technicians have the role of visiting schools and educating children about rabies. Among other resources, they utilize a government prepared video entitled “If I Only Knew” and various informative pamphlets discussing rabies. We found that schools and school children accounted for 19% of the population's knowledge source across the province. However, there was not a significant relationship between the presence of school children and knowledge of rabies in individual households (χ^2^ = 0.027, df = 1, *p = *0.868).

**Figure 3:**
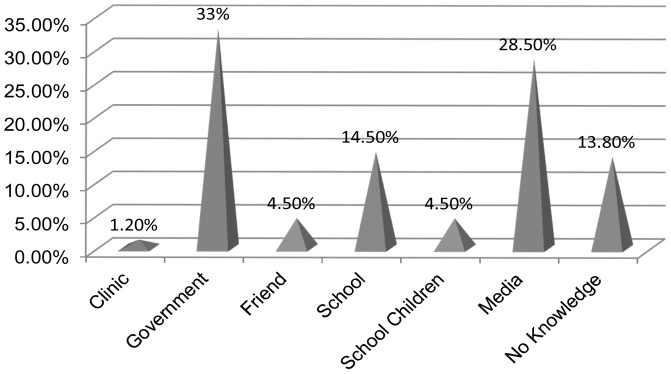
Source of rabies knowledge reported by both dog owners and non-dog owners across KwaZulu-Natal (n = 1982).

Less than two percent of the surveyed population indicated that they have acquired rabies knowledge from the local health clinic. Since one objective of our research was to understand where citizens were able to receive valuable information about rabies, any viable source was noted and utilized as an element of feedback for the development of future programs employed by the government departments responsible for human and animal health. Public print and broadcast media were cited highest among non-dog owners as their source of rabies knowledge, whereas government sponsored campaigns were most frequently cited from dog owners (Figure 3).

### Dog bite histories

12.7% (95% CI 11.3–14.2) of the 1992 households in the areas surveyed reported that at least one member of the household had been bitten by a dog in the past year. Age of bite victim was not recorded. The lowest incident rate was in Esikhawini (urban) and the highest incidence occurred in Wembezi (peri-urban). Across KZN, significantly more people who did not own dogs had been bitten by a dog than those who did own a dog (χ^2^ = 9.477, df = 1, *p = *0.002). Among victims who were dog owners the number of dogs owned did not make a difference in dog bite incidence. Although 33% (667/1992) of households reported feeding of dogs that they did not own (on their property), there was not a significant relationship between being bitten by a dog and feeding other dogs on the property (χ^2^ = 3.424, df = 1, *p* = 0.064).

**Figure 4:**
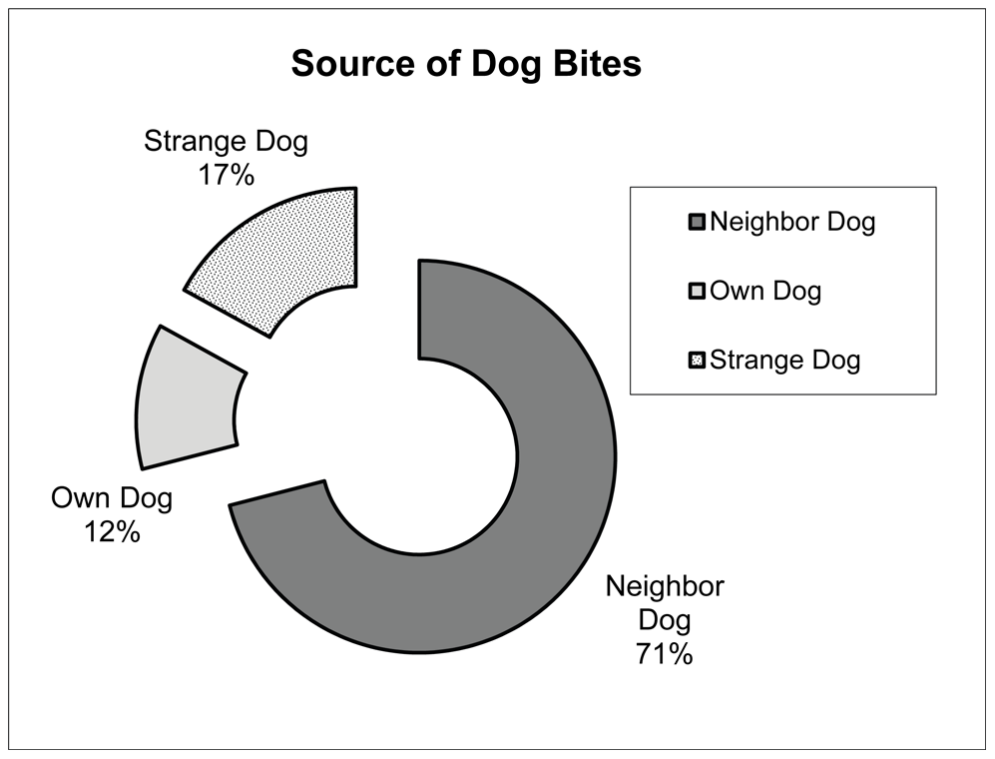
Dogs identified by victims bitten in bite incidents within a one year period (2011) across KZN (n = 227).

As a follow up question, households with recent dog bite victims were asked if they knew the aberrant dog involved in the incident (Figure 4). Identification of the offending dog was missing in 10% (26/253) of recorded cases. In 71% of records where the dog was identified, the neighbor's dog had bitten the victim. Only 12% of victims had been bitten by their own dog and 17% of victims had been bitten by a dog with which they were unfamiliar. Unknown dogs were referred to as strange dogs rather than stray dogs, as the animal could be owned but unrestricted.

### Response to bite

Historically there have been concerns about people in rural areas visiting traditional healers rather than attending a clinic after a dog bite event. Sudarshan et al [15] showed that 60% of dog bite victims in India who succumbed to rabies had sought some kind of indigenous treatment following the incident, receiving either magico-religious practices or some kind of herbal therapy. After a recent emerging rabies epidemic in the Limpopo province of South Africa (2005–2006), it was established that 20% of the fatal human rabies case patients saw a traditional healer prior to attending hospital [16]. In this survey in KZN, less than 2% (4/253) of dog bite victims, all of whom were from rural areas, reported resultant visits to a traditional healer.

With regard to the washing of bite wounds as a first response to incident, only 8% of victims mentioned washing the wound. We found that 56% (1115/1992) of domiciles visited in our surveys had either a pit latrine or no toilet facilities, indicative of a lack of running water at the household level. In some rural areas a public tap was available some distance from the house. In other rural areas, people made use of rivers or streams for daily water.

**Figure 5:**
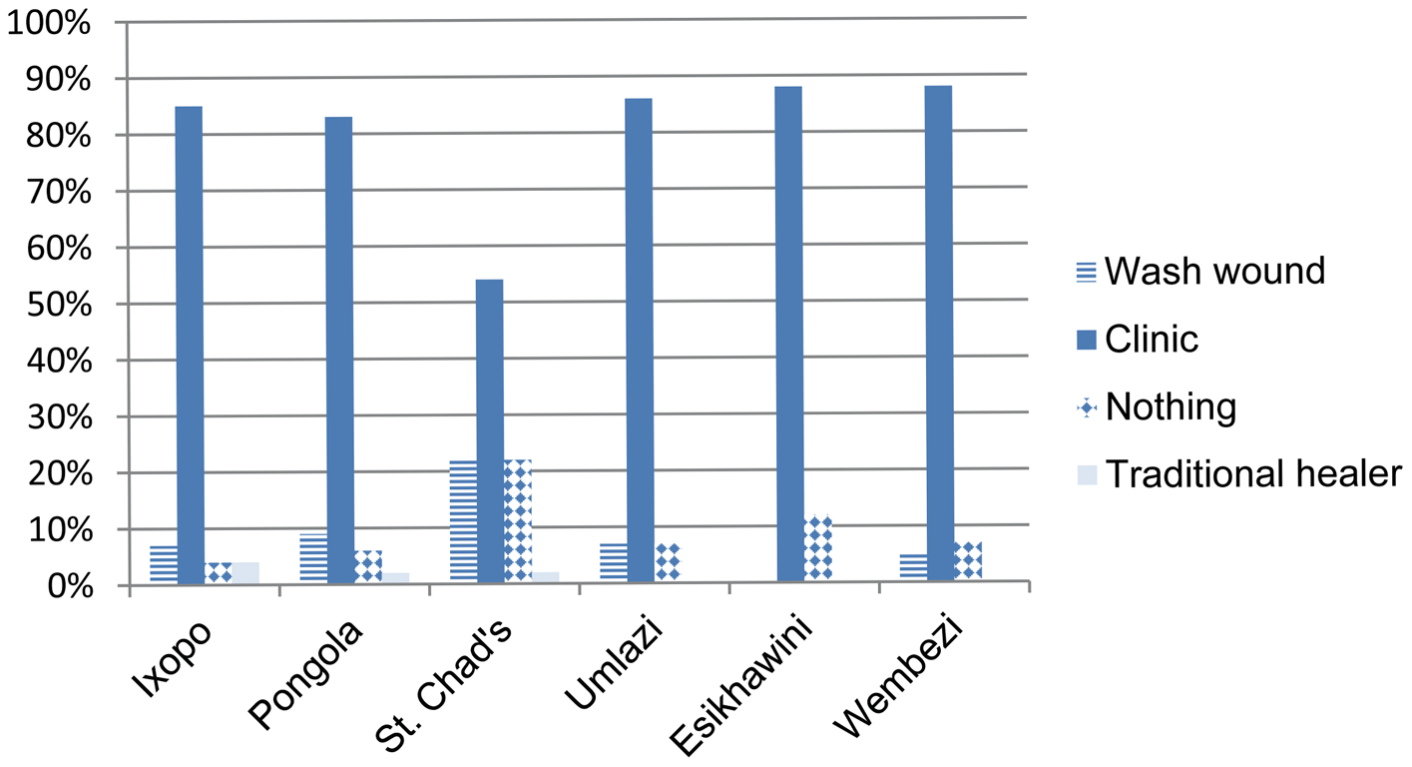
Treatment of bite by area surveyed (n = 227). (Rabies enzootic rural = Ixopo, Pongola, St. Chad's; rabies enzootic urban = Umlazi, Esikhawini; rabies free peri-urban = Wembezi).

In the six areas surveyed, over 80% (207/253) of victims visited a clinic as a response to dog bite incident except in the rural area of St. Chad's (Figure 5). This area alleged the highest rate of rabies knowledge (88%) (Figure 2), but had the least number of visits to the clinic (54%) as a response to bite incidence. St. Chad's has both a community health center and close access to neighboring community clinics and hospitals. Detailed questions that would uncover the decisions made by bite victims, actions taken and reasons for those actions were not asked.

### Prophylaxis received at clinics

**Figure 6:**
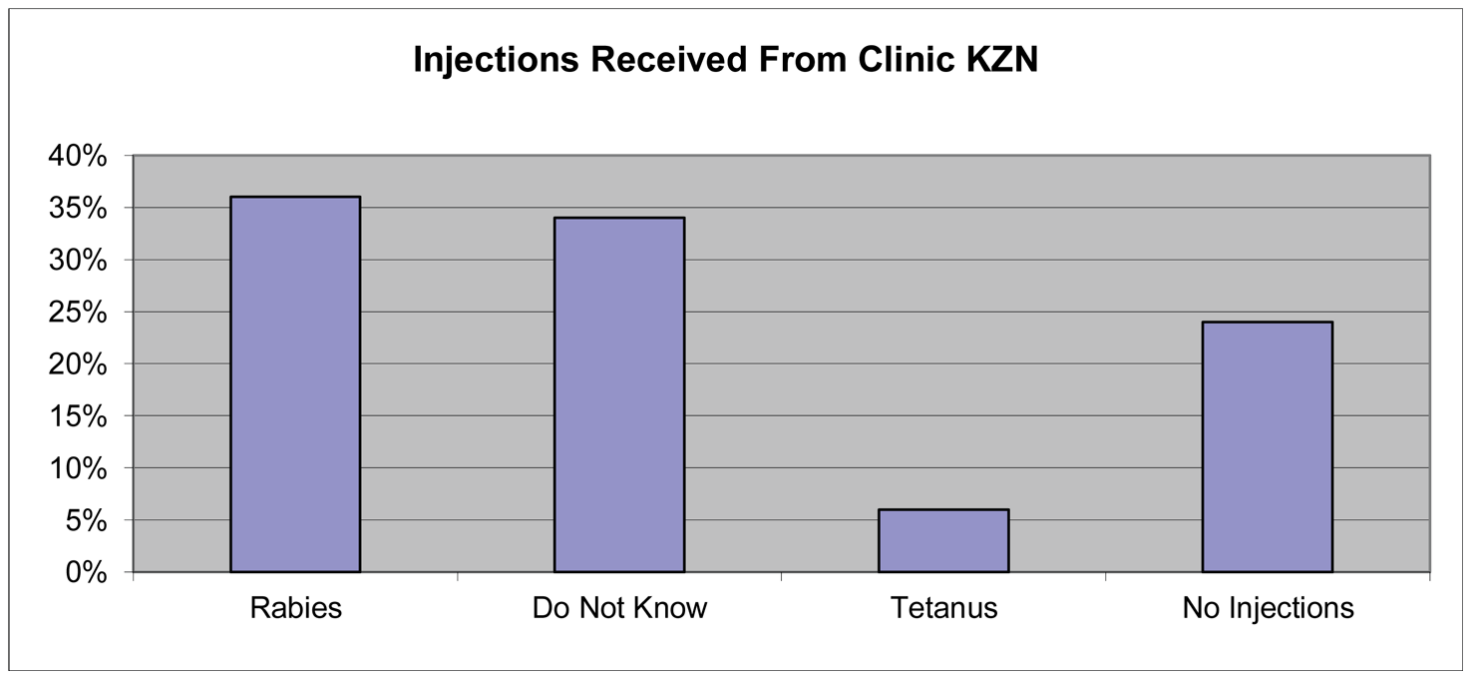
Injections identified as received by clinic patients across the province based upon respondent recall (n = 207).

Those households with victims who had visited a clinic in response to a dog bite were asked what injections they had received (Table 1, Figure 6). Twenty-four percent (50/207) of clinic visitors reported receiving no injections. Thirty-four percent (70/207) of respondents did not know the extent of treatment received, as it was either not explained to them, they could not remember or they did not attend the clinic with the victim. Thirty-five percent (73/207) of persons visiting the clinic received rabies vaccine, 5% received tetanus only and 1.4% received both rabies and a tetanus vaccine. Those victims who said they received rabies vaccine were not asked if they returned to the clinic to complete the World Health Organization recommended (Essen schedule in the case of South Africa) four injection series or if they received immunoglobulin in the case of category III bites [17]. Severity and location of bite wounds was not queried of respondents.

**Table 1:** Distribution of dog bites, offending dog and clinic visits resulting in post-exposure rabies vaccine.

Area Surveyed	Dog Bite last 12 months	Own Dog	Neighbor Dog	Strange Dog	Clinic Visits	Received Rabies Vaccine
Esikhawini	32 (10.06%)	1	24	7	28 (88%)	21 (75%)
Ixopo	47 (14.07%)	6	28	6	40 (85%)	16 (40%)
Pongola	48 (13.87%)	3	34	4	40 (83%)	14 (36%)
St. Chad's	41 (11.48%)	7	25	5	22 (54%)	7 (32%)
Umlazi	42 (12.6%)	3	24	11	36 (86%)	8 (22%)
Wembezi	43 (14.33%)	6	27	6	38 (88%)	10 (26%)

### Rabies prophylaxis model

An effort was made to determine if there was an association between those clinics where patients had received rabies vaccine and the area surveyed, whether the victim owned dogs, respondent knowledge of rabies and identification of offending dog. Fifty-two responses (20%) were deleted from the model due to missing values for either the response or explanatory variables. In the final logistic regression model only the area surveyed significantly contributed to human rabies vaccination outcome (Table 2).

**Table 2:** Effects variables used in forward selection for logistic regression model predicting human rabies PEP**.

Analysis of Effects Eligible for Entry			
Effect	Degrees of Freedom	Chi Square	Probability
Area	5	24.8665	0.0001*
Own Dogs	1	0.0301	0.885
Know Rabies	1	0.4254	0.3978
Bitten By	2	1.6977	0.2021

* Level of significance necessary for entry into the model was 0.05.

** Post-exposure prophylaxis.

The model appeared to fit the data (Somer's D = 0.395). The urban township of Esikhawini was the area with the most dog bite victims receiving rabies vaccine, while victims in urban Umlazi Township received the least (Table 3).

**Table 3:** Results of effects model indicating areas with patients most and least likely to receive PEP.

Analysis of Maximum Likelihood Estimates					
Parameter	Degrees of Freedom	Estimate	Standard Error	Wald Chi Square	Probability
Intercept	1	−0.6771	0.1612	17.6312	<0.0001
Esikhawini	1	1.419	0.3527	16.1847	<0.0001
Ixopo	1	−0.0161	0.3208	0.0025	0.9601
Pongola	1	0.4088	0.3413	1.4346	0.231
St. Chad's	1	−0.7092	0.3809	3.4677	0.0626
Umlazi	1	−0.9323	0.3992	5.4555	0.0195
Wembezi	1	−0.1702	0.3631	0.2198	0.6392

By estimate value, patients in Esikhawini were most likely to receive rabies vaccine.

### Interest in zoonoses

Respondents were queried if they were interested in learning more about illnesses that could be shared between people and animals. Ninety-four percent of the population surveyed across the province (1865/1992) said they would be interested in gaining information about zoonotic disease potential in their community. The urban area of Umlazi, where only 88% of the respondents answered agreeably, stood out as the only area where there are a significant number of respondents who were disinterested in zoonoses (χ^2^ = 30.581, df = 10, *p* = 0.001).

### Community based surveillance

**Figure 7:**
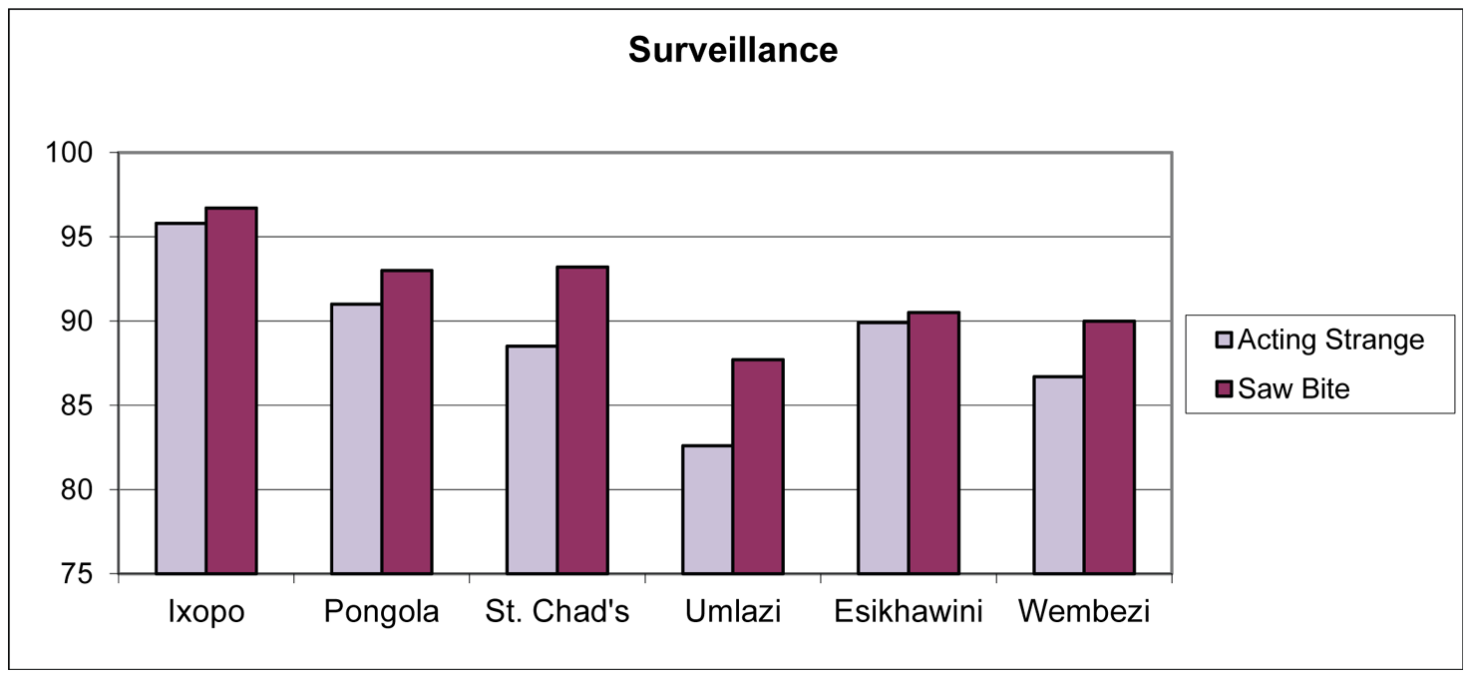
Interest in surveillance activities by community across the province (n = 1989). (Rabies enzootic rural = Ixopo, Pongola, St. Chad's; rabies enzootic urban = Umlazi, Esikhawini; rabies free peri-urban = Wembezi).

Respondents were asked if they would, as witnesses, be interested in reporting ill dogs, strange behavior or dog bite incidents occurring in their communities. The majority of respondents across all communities were interested in reporting such sightings; however, the peri-urban and urban communities had significantly less interest than the rural areas in community based reporting (χ^2^ = 22.120, df = 5, *p* = 0.000) (Figure 7).

**Figure 8:**
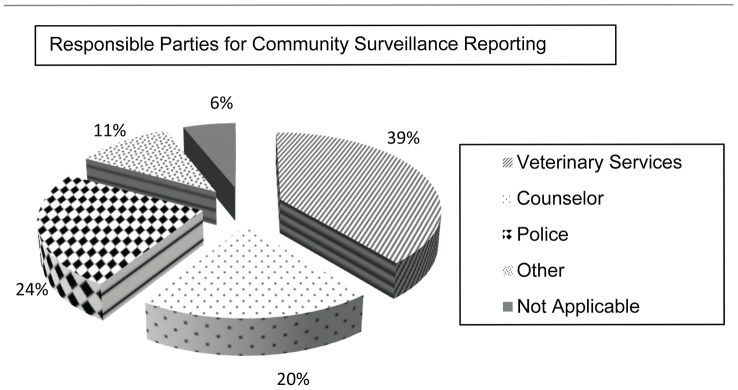
Responsible parties identified by respondents for reporting community based surveillance activities across the province.

Respondents answering in the affirmative were further queried as to whom in the community they would want to report these incidents. Nearly 40% identified government veterinary services when considering reporting sick dogs and possible rabies cases (Figure 8). Other parties mentioned were the local clinic, a teacher, the dog's owner, or SPCA. However, community members regularly stated that despite their desire to report, they had no contact information for either veterinary services or the SPCA.

### Area wildlife sightings

**Figure 9:**
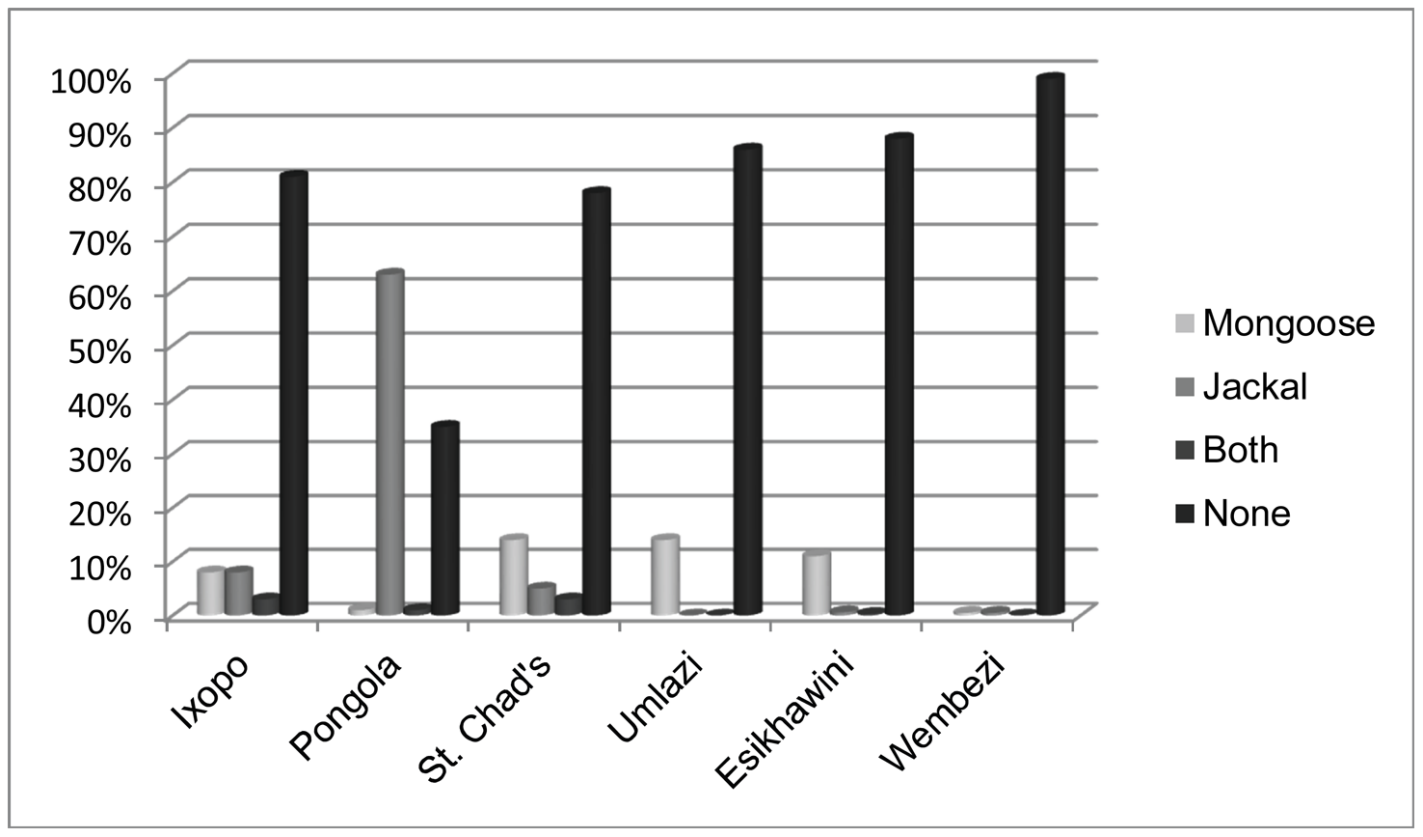
Wildlife sightings across areas surveyed (n = 1990). (Rabies enzootic rural = Ixopo, Pongola, St. Chad's; rabies enzootic urban = Umlazi, Esikhawini; rabies free peri-urban = Wembezi).

Other than dogs, 2 potential rabies maintenance hosts present in KZN are mongooses and jackals. Bat eared foxes, which maintain rabies in the western provinces of South Africa [18], are rarely seen in KZN. Questioning respondents about sightings in their community served as a screening tool for the possibility of further studies concerning wildlife and the spread of rabies in KZN. Overall, less than 22% of the respondents across the province encountered either of these wildlife species in their communities (Figure 9). The rural tribal authority area outside of Pongola has dense flora and is located on the Swaziland border which could explain why there were so many more jackal sightings in this rural area versus any other.

### Animal control laws and regulations

**Figure 10:**
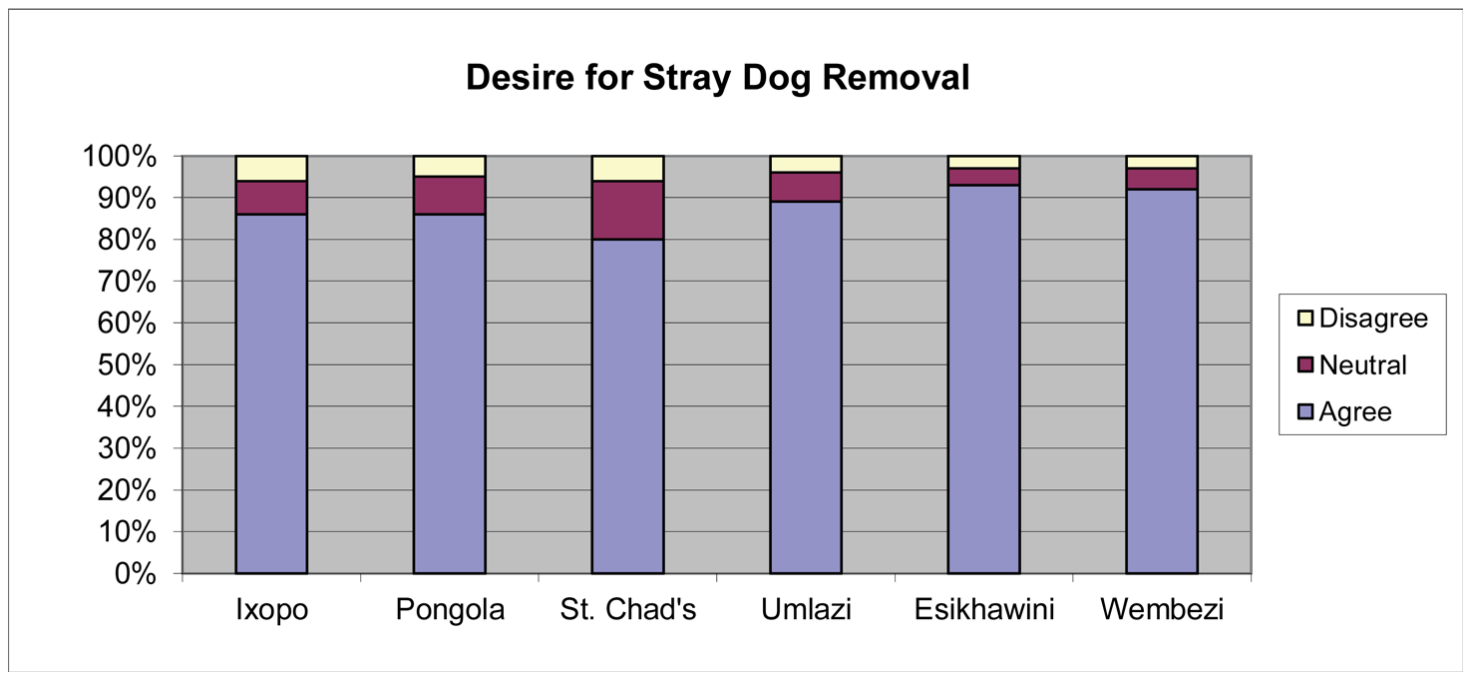
Desire for dog control by area surveyed (n = 1984). (Rabies enzootic rural = Ixopo, Pongola, St. Chad's; rabies enzootic urban = Umlazi, Esikhawini; rabies free peri-urban = Wembezi).

Despite South Africa possessing laws requiring vaccination and licensure of dogs, these regulations are rarely enforced. Respondents were asked if they desire law enforcement regarding removal of stray or unsupervised dogs (Figure 10). There was a significant difference in area type and desire for animal control law enforcement, with the least concern reported from the rural areas (*p* = 0.0001).

**Figure 11:**
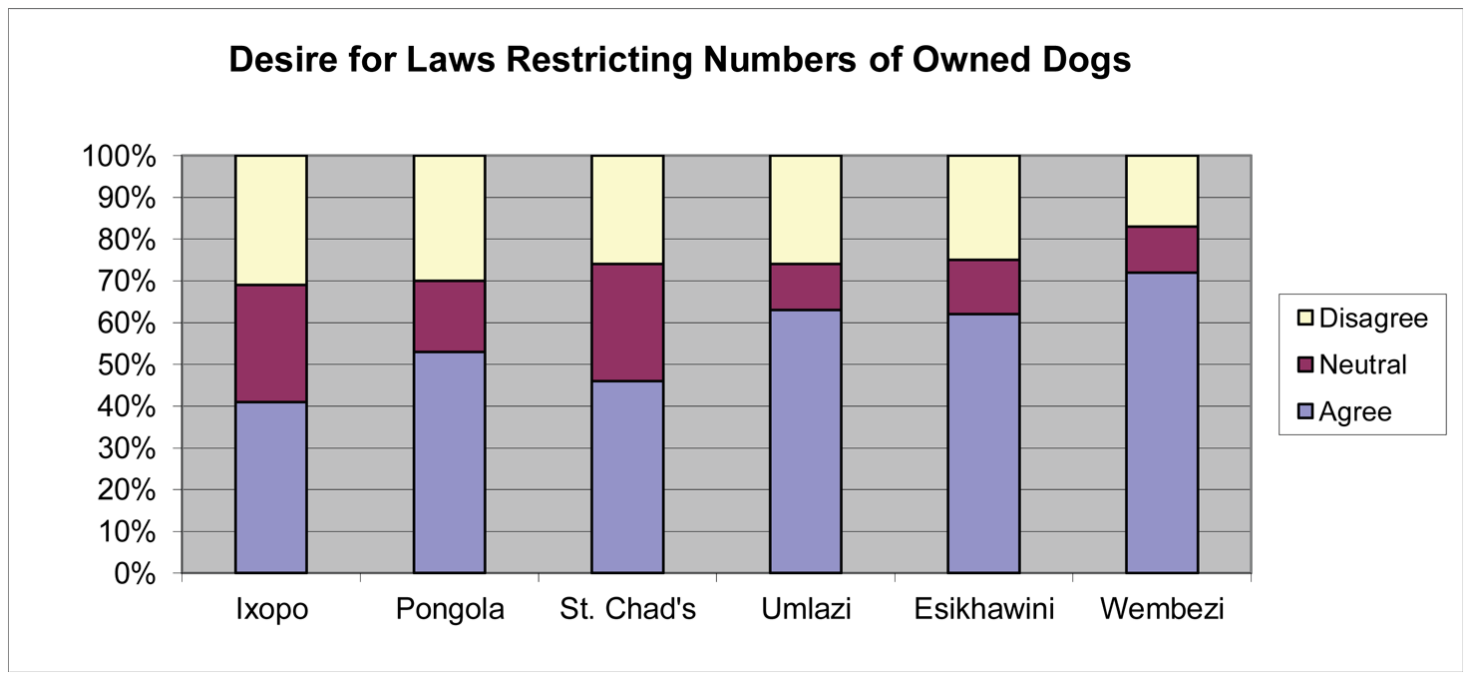
Respondent desire for laws imposing limitations on dog ownership (n = 1988). (Rabies enzootic rural = Ixopo, Pongola, St. Chad's; rabies enzootic urban = Umlazi, Esikhawini; rabies free peri-urban = Wembezi).

Surveys respondents were asked if they desired laws that would limit the number of dogs that one household could own (Figure 11). The number of dogs owned by the dog owning households surveyed was similar between the rural and peri-urban households with an average of 2.47 dogs per dog owning household (range 2.16–2.64). Urban dog owning households had fewer dogs with the average being 1.66 (range 1.64–1.68). Some households in rural areas were recorded as owning up to 19 dogs. There was a significant difference between rural areas and the urban/peri-urban areas in desire for limitations on the number of dogs one household could own (*p* = 0.0001).

## Discussion

The results from this survey indicate that 86% of persons in high risk canine rabies areas of KwaZulu-Natal have at least heard of the disease even if they are unaware of the details surrounding transmission and consequences of exposure. The long history of enzootic canine rabies in the province and the continuous efforts put forth by the KZNDAERD- Government Veterinary Services appear to contribute the most (33%) to this public awareness. Although 100% would be ideal, an 86% knowledge rate is better than was reported from other studies. In dog rabies enzootic Zimbabwe, for example, 74% of pet owners interviewed in Harare animal clinics were aware that rabies was transmitted to people by dogs [19]. Zimbabwean respondents also reported gaining information about other zoonotic diseases from their veterinarian. Respondents in KZN do not have local veterinary clinics as a resource from which to gain this knowledge. In the current study, the peri-urban working community of Wembezi had the lowest rate of rabies knowledge at 81%. This is interesting in that it is also the only community identified as being free of canine rabies for greater than 10 years per surveillance records from KZNDAERD. Thirty-nine percent of households in Wembezi owns at least one dog and has an estimated dog population of 2, 916 (Hergert unpublished data). Calculated dog density figures for Wembezi are similar to the urban areas surveyed in this study, where a slightly higher level of rabies knowledge was recorded. Non-dog owners were 1.6 times more likely to have heard of rabies versus dog owners. People who do not own dogs are gaining information about rabies from media sources, which had a value of almost 30% from respondents in all areas. This is similar to findings in the developed world – e.g. in Texas, USA 43% of non-pet owners reported learning about zoonotic diseases from the media or newspaper [20]. Media sources for rabies information may actually be viewed by both the dog owning and non-dog owning public; however, dog owners may be more likely to respond that their source of rabies knowledge was from government vaccination campaigns, as they would be attending these events. Therefore, a certain amount of bias may weigh towards government campaigns as a source for dog owners due to their familiarity. Understanding why more non-dog owners report knowing about rabies versus dog owners is unclear from this survey and would require further study. South African Government Veterinary Services has the task of informing people about rabies through vaccination campaigns and schools. When these two reported sources of knowledge are combined it is evident that Veterinary Services is responsible for 52% of the information gained by both the dog and non-dog owning public. However, there was not a significant relationship between household knowledge of rabies and knowledge source originating from schools. Veterinary Services of KZN might take into consideration when planning educational campaigns in their communities, that schools are not heavily targeted. Eighty-two percent of interviewed households contained school aged children; therefore, schools appear to be a viable outlet for the dissemination of rabies information. Human health clinics were reported as a knowledge source in less than 2% of responses. This result may support the findings from Francophone countries of Africa where medical authorities and health practitioners are reported to be under educated in the perils of rabies [5]. In Texas, USA, the family doctor was reported as the source of zoonotic disease information in only 6% of both pet and non-pet owning households [20]. Doctors were also indicated well below veterinarians and the media as a source of disease information in Zimbabwe [19]. Detailed examination into what transpires in KwaZulu-Natal clinics for dog bite case patients should be explored in the face of a One Health environment.

Twelve percent of the households in the areas surveyed had someone bitten by a dog in the last year. Other animal bite victims in African countries have been identified through retrospective studies starting at the clinic or hospital level using a trace back system in order to locate and interview the victims in depth [21]–[22]. This type of retrospective study should be conducted in KwaZulu-Natal in order to gain further descriptive information of the dog bite incidence.

The neighbor's dog was identified as the offending canine in 71% of bite cases in this survey. Only 12% of the people had been bitten by their own dog. However, 17% of victims had been bitten by a dog with which they were unfamiliar. These dogs were identified as strange rather than strays or feral dogs, as they could not be differentiated from owned free roaming dogs. Eighty-three percent of dogs in this study were identified as being fully or partially unrestricted, being allowed to wander at will. Over 96% of the human rabies cases in India from 1992 to 2002 resulted from a dog bite, with 75.2% resulting from stray dogs and 11.1% from pets [15].

In response to the dog bites more than 80% of people went to the clinic in all areas except for rural St. Chad's where only 54% of bite victims reported clinic visits. St. Chad's residents reported a high awareness of rabies (88%), which could be explained by the rabies epidemic experienced in the area a few months prior to the survey. This area has a community clinic, as well as other nearby clinics and hospitals reachable by taxi. Without in depth queries, the reason why more persons did not visit a clinic remains unknown. Despite concerns about delayed treatment after dog bites, less than 2% of victims in this study visited a traditional healer and all of those cases were from rural areas. Herbal therapy and magico-religious practices were sought by rabies bite victims in India in 60% of fatal cases [15]. Respondents in KZN may be more informed about rabies than persons in other developing countries. One survey respondent said that the reason he did not go to the clinic after being bitten by his neighbor's dog was because the neighbor could prove to him that his dog had been previously vaccinated against rabies. Therefore, the victim felt he was safe to treat the wound at home. The investigation, follow through and cognition shown by this respondent is not something that should be expected from most bite victims. Dog bite victims in this study tended to visit the clinic regardless of their familiarity with the dog that bit them.

Of those victims that did attend a clinic 22–75% received at least one rabies vaccine. The lower end of this spectrum is similar to what was seen in India where only 21% of rabies victims had received at least one rabies vaccine [15]. The area with the lowest rabies vaccination treatments was urban Umlazi Township and the highest was urban Esikhawini Township. In the regression model predicting what factors had an important impact on the victim receiving a rabies vaccine, only the area surveyed was found to be significant (*p* = 0.0001). Health facilities in South Africa where rabies vaccine and immunoglobulin (RIG) are available are listed with telephone contact numbers in the national rabies guideline [23]. However, a nationwide telephonic survey, which included 50% of the facilities identified for KwaZulu-Natal, was conducted in order to confirm the availability of these products. Only 68% of all the sites surveyed across the country were contactable by telephone. Forty-one percent had both vaccine and RIG, 32% had only vaccine, 5% had only RIG and 21% had neither vaccine nor RIG available [24]. Considering the results of this telephonic survey it is quite conceivable that administration of rabies vaccine is area dependent across the country. The juxtaposition of Esikhawini Township to the Port of Richard's Bay could explain why this area, which had the lowest recorded number of dog bite cases, had the highest amount of rabies vaccine administered. The Richard's Bay area may have more rabies vaccine dispensed that are related to aspects about the constituents the medical community serves, or because the medical staff could be indiscriminately dispensing supplies regardless of exposure risk.

In Francophone African countries accurate rabies data is scarce [5]. This may be true in other African countries as has been previously eluded from Tanzania [1]. This survey showed a large respondent willingness to participate in community based surveillance at the village level. Community based surveillance activities should be considered in countries which lack central political will or local municipal finances. However, it has been stated that passive systems in developing countries are ineffective; therefore, an economic community based active surveillance system is recommended [25]. Unfortunately, community based systems have been shown to fail, particularly when there is a discrepancy in the interpretation of needs between the community and the donor organization [26]. Therefore, the methodology to be employed would have to be developed from a grassroots level rather than at a higher administrative level, which would take a commitment not previously demonstrated from this rank of society.

Persons living in communities at high risk for canine rabies are interested in animal control laws and regulations. However, there is an indication of concern in the rural areas that these laws would also limit the number of livestock owned. This result may be due to rural areas owning more livestock. An indirect association between the limitations on number of dogs allowed with restrictions on livestock ownership may be behind these results. In rural Texas, USA, a survey regarding cattle ownership conducted from Texas A&M University indicated that ranchers were reluctant to comply with trace back ear-tagging measures, as the procedure would identify to officials how many cattle were owned by each producer at any point in time (Dominguez unpublished data). Responsiveness and dedication to upholding animal control laws in this cultural environment by obliged parties will have to be instilled in a generation of officers committed to uplifting the community.

Crucially, since this simple intervention may be particularly effective in preventing infection, only 21 of the 253 people in our survey bitten by a dog washed their bite wound as a response to treatment. It has been established that washing the bite wound for 15 minutes with soap and water can help reduce the incidence of disease by eliminating or inactivating the virus [5]. As only 44% of households were reported as having indoor plumbing (indicated by flush toilets) lack of available fresh water may explain the low percentage of wound washing as a response to post-bite treatment.

## Conclusions

This study shows that greater than 86% of the population has at least heard of the disease called rabies, but the response to dog bites indicates that both the general public and health sectors of the population do not understand the possible consequences related to dog bites in rabies enzootic environments. Availability of vaccine is an important factor in determining if bite victims receive rabies vaccine during clinic visits in KwaZulu-Natal and other parts of Africa; however, factors within the clinic setting such as staff knowledge need to be considered as well. Consideration of the offending dog in the bite incident has not been shown to play a role in victim response to dog bites. Therefore, the wasting of PEP could be as a big a problem as people at risk not receiving necessary vaccine. Our results also indicate that schools and rabies education of schoolchildren can be much improved. Not only are children most at risk of rabies exposure, but schools may present appropriate structures for dissemination of this kind of information and should be utilized to a greater extent. Questions in this survey regarding response to dog bites could have been more detailed. An example would be to include the age of the bite victim as a variable. Regardless, these results lend credence to the statement that an in-depth study regarding the treatment people are receiving and the public knowledge of rabies needs to be conducted.

## Supporting Information

Checklist S1 Strobe Checklist.
(DOC)
